# Understanding Bidirectional Water Transport across Bronchial Epithelial Cell Monolayers: A Microfluidic Approach

**DOI:** 10.3390/membranes13120901

**Published:** 2023-12-06

**Authors:** Miroslaw Zajac, Slawomir Jakiela, Krzysztof Dolowy

**Affiliations:** Department of Physics and Biophysics, Institute of Biology, Warsaw University of Life Sciences, 02-776 Warsaw, Poland; slawomir_jakiela@sggw.edu.pl

**Keywords:** bronchial epithelial cells, water transport, ion transporters, bidirectional flow, microfluidic droplet system

## Abstract

Deciphering the dynamics of water transport across bronchial epithelial cell monolayers is pivotal for unraveling respiratory physiology and pathology. In this study, we employ an advanced microfluidic system to explore bidirectional water transport across 16HBE14σ bronchial epithelial cells. Previous experiments unveiled electroneutral multiple ion transport, with chloride ions utilizing transcellular pathways and sodium ions navigating both paracellular and transcellular routes. Unexpectedly, under isoosmotic conditions, rapid bidirectional movement of Na^+^ and Cl^−^ was observed, leading to the hypothesis of a substantial transport of isoosmotic solution (145 mM NaCl) across cell monolayers. To validate this conjecture, we introduce an innovative microfluidic device, offering a 500-fold sensitivity improvement in quantifying fluid flow. This system enables the direct measurement of minuscule fluid volumes traversing cell monolayers with unprecedented precision. Our results challenge conventional models, indicating a self-regulating mechanism governing water transport that involves the CFTR channel and anion exchangers. In healthy subjects, equilibrium is achieved at an apical potential of Δφ_ap_ = −30 mV, while subjects with cystic fibrosis exhibit modulation by an anion exchanger, reaching equilibrium at [Cl] = 67 mM in the airway surface liquid. This nuanced electrochemical basis for bidirectional water transport in bronchial epithelia sheds light on physiological intricacies and introduces a novel perspective for understanding respiratory conditions.

## 1. Introduction

In our prior investigations, we unveiled the intricate dynamics of ion transport across 16HBE14σ cell monolayers. It emerged that chloride ions (Cl^−^) traverse through transcellular pathways, whereas sodium ions (Na^+^) navigate both paracellular and transcellular routes. Remarkably, there was an absence of transmembrane potential fluctuations during this ion transport, rendering the process electroneutral.

Surprisingly, the swift movement of Na^+^ and Cl^−^ across the cell monolayer occurred bidirectionally, even under isoosmotic conditions on both sides of the cellular barrier. Our astute observations led us to hypothesize a voluminous transport of isoosmotic solution (145 mM NaCl), roughly 5 µL, across 16HBE14σ cell monolayers within a mere one-minute timeframe [1,2,3]. Seeking validation for our conjecture, we conducted direct measurements of fluid flow across cell monolayers, mirroring the conditions of our multiple ion transport assessments.

Despite the remarkable advancements in organ-on-a-chip models [4,5,6] the precise measurement of fluid transport across the epithelium remains a formidable challenge [7,8]. The airway epithelium plays a pivotal role as a barrier, encompassing chemical, physical, and immunological functions that effectively separate the internal milieu from the external environment [9]. It is lined by a thin layer of airway surface liquid (ASL) generated by fluid secretion from surface epithelial cells and submucosal glands. Maintaining ASL homeostasis is crucial for proper lung function and requires tight regulation of its composition, volume, and pH [10]. The direction of water transport across the epithelium is determined by transcellular and paracellular movements of ions and water, primarily driven by transport of sodium, potassium, chloride, and bicarbonate [11].

The initial endeavor to indirectly determine water transport was pioneered by the Danish duo, Nobel Prize recipients George von Hevesy and August Krogh, who employed D_2_O as a tracer [12]. Although the use of radioactive water (D_2_O or T_2_O) became widespread, it necessitated safety precautions and was susceptible to experimental errors [13,14,15].

Directional water flux across epithelia triggers an extensive exchange of water molecules between the epithelial layers. The simplistic diffusion model falters in the face of unstirred layers, where the tracer concentration diverges from the bulk solution. Another indirect method, utilizing a fluoresce indicator, measures fluid transport by tracking changes in fluorescence under a confocal microscope [16]. A miniaturized adaptation of this method was employed to study fluid transport variations across epithelia grown on porous supports subjected to osmotic gradients [17]. Notably, the use of fluorescent tracers introduces drawbacks such as the photobleaching phenomenon and interactions with living cells.

Direct fluid transport measurement methodologies have also evolved. In an early initiative, Wiedner (1976) utilized a capacitance transducer positioned above the medium surface enveloping the epithelium, sealed between two Ussing hemi-chambers [18]. Fluid transport was deduced from the subtle rise in the medium level. However, the sensitivity of this method to biological impurities and its susceptibility to shape changes in the meniscus at the medium–air interface posed challenges. An alternative method involved two chambers separated by a folded, impermeable plastic membrane. One chamber, connected to the Ussing system, terminated with a capillary, and the movement of the opaque fluid meniscus in the capillary was detected optically [19]. Yet, a significant drawback of this approach was the substantial surface tension at the interface between the solution and air in the capillary, obscuring the undisturbed flow of the medium.

In light of these challenges, we opted for a direct measurement of fluid flow across epithelial cell line monolayers through a microfluidic system employing an oil drop within the water column of the measuring capillary [20,21]. Our innovative measurement device boasts a sensitivity 500 times greater than previous direct flow determination methods. This groundbreaking advancement has, for the very first time, empowered us to precisely quantify minuscule volumes of fluid traversing a monolayer of cells cultivated on a porous substrate, spanning a mere 1.12 cm^2^.

## 2. Materials and Methods

### 2.1. Cell Culture

The 16HBE14σ cell line, derived from the human bronchial epithelium and graciously provided by Dr. Dieter Gruenert of UCSF, was cultivated following established protocols [1,2,3]. In brief, the cells were cultured in Minimum Essential Medium (MEM) from Merck and supplemented with 10% fetal bovine serum (FBS) from Gibco and 1% penicillin/streptomycin from Merck. The cultivation took place in T75 cell culture flasks (Nunc), with cells being passaged upon reaching 70–90% confluence.

Subsequently, the cells were seeded onto Snapwell inserts (Corning Costar) at a density of 2.5 × 10^5^ cells/cm^2^. A liquid–liquid interface was maintained for an initial period of 7–9 days, followed by transition to an air–liquid interface for an additional 14–16 days to induce cell polarization. To ensure the quality of the cell monolayers, their electrical resistance was measured both before and after each experiment using an EVOM device from WPI. Cell layers with a resistance below 300 Ωcm^2^ were excluded from subsequent analysis.

### 2.2. The Media

In the investigation of water transport, Krebs–Henseleit solution (KHS) and its two modifications, differing in sodium and chloride concentrations, were employed. The compositions of the solutions were as follows:

- KHS1 (physiological Na^+^ and Cl^−^) contained 117 mM NaCl, 4.7 mM KCl, 2.5 mM CaCl_2_, 1.2 mM MgCl_2_, 1.2 mM KH_2_PO_4_, 24.8 mM NaHCO_3_, 11.1 mM glucose;
- KHS2 (referred as “low sodium” solution) contained 4.7 mM KCl, 117 mM choline chloride, 4.7 mM KCl, 2.5 mM CaCl_2_, 1.2 mM MgCl_2_, 1.2 mM KH_2_PO_4_, 24.8 mM NaHCO_3_, 11.1 mM glucose;
- KHS3 (referred as “low chloride” solution) contained 10 mM NaCl, 107 mM Na-gluconate, 1.2 mM KH_2_PO_4_, 24.8 mM NaHCO_3_, 4.7 mM K-gluconate, 1.2 mM Mg-gluconate, 2.5 mM Ca-gluconate, and 11.1 mM glucose.

To maintain a consistent pH of 7.35, the solutions underwent pH adjustment with NaOH/HCl, followed by bubbling with carbogen (95% O_2_ and 5% CO_2_) until reaching a final pH of 7.4. It is noteworthy that all three solutions maintained an identical osmotic pressure (Π = 296 ± 6 mOsm/kg H_2_O).

## 3. Precision Microfluidics for Controlled Droplet Generation and Velocity Measurement in Epithelial Cell Studies

### 3.1. Experimental System Design Objectives

**Figure 1:**
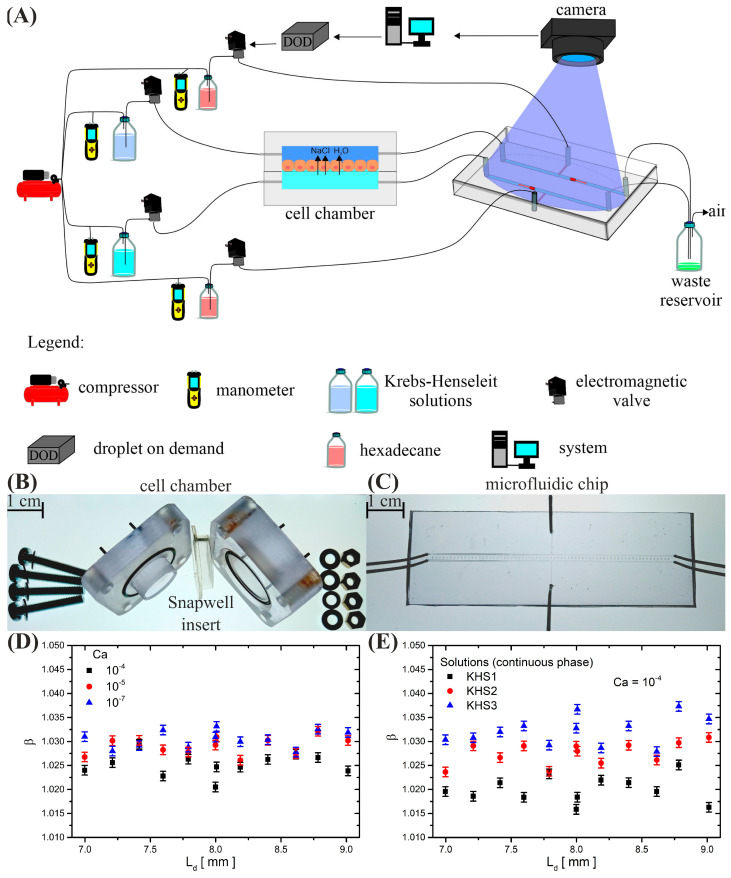
(**A**) A comprehensive illustration of the measurement system is presented, with detailed descriptions provided in the legend. (**B**) The physical manifestation of the polycarbonate chamber, designed to house the epithelial cell insert, is depicted. (**C**) An actual representation of the microfluidic chip, featuring two T-arms designed for measuring the mobility of an oil droplet within a KHS fluid environment. (**D**) A graphical representation illustrating the relationship between oil-droplet mobility and length for three distinct capillary numbers. In this specific experiment, the continuous phase was represented by KHS1. (**E**) A graphical depiction of the dependence of oil-droplet mobility on capillary numbers $\;Ca=10^{-4}$ across different continuous phases: KHS1, KHS2, KHS3. (Mobility (β) is defined as the velocity of the droplet phase relative to the average velocity of the continuous phase.)

The experimental system (see Figure 1A) was designed with three primary objectives: (i) to minimize the necessity for human intervention during measurements, (ii) to reduce fluctuations in temperature within the liquids and the system, and (iii) to optimize for minimal uncertainty in droplet velocity measurements.

### 3.2. Fabrication of Epithelial Cell Chamber and Microfluidic Chip

The epithelial cell chamber (see Figure 1B) was meticulously milled into 10 mm thick polycarbonate plates (Makrolon, Bayer, Leverkusen, Germany) using a precision milling machine (MSG4025, Ergwind, Gdansk, Poland). Two asymmetrical halves (4 × 4 cm) were crafted with shapes tailored to Snapwell (Corning Inc., Costar, NY, USA) inserts (see Figure 1B), ensuring a fixed volume of 20 μL for both the apical and basolateral chambers. These halves were sealed with two O-rings. The integration of two O-rings impeccably sealed these halves, promoting a robust and hermetic enclosure. The assembly of the chamber was completed by securely fastening the two halves with M3 screws. This design not only facilitated the effortless replacement of the cell insert but also empowered the execution of excimer experiments within the same geometric configuration. The utilization of M3 screws, in particular, exemplified our commitment to experimental versatility and the pursuit of scientific rigor in our investigations.

Simultaneously, a polycarbonate microfluidic chip (see Figure 1C) was fashioned from 3 mm thick plates (Makrolon, Bayer, Leverkusen, Germany) through CNC milling (MSG4025, Ergwind, Gdansk, Poland). Subsequently, the two milled plates of the microfluidic system were thermally bonded by compressing them together at 130 °C for 30 min [22]. The cross-section of all channels was standardized at 400 μm × 400 μm. Surface modifications, following protocols described by Jankowski et al. [23], were applied to facilitate oil-in-water droplet generation. The dispersed phase comprised hexadecane (H6703, Sigma-Aldrich, Saint Louis, MO, USA) within a continuous water phase.

Inlets and outlets for both the cell chamber and microfluidic chip were facilitated by 10 steel needles (~4 cm long, O.D. 0.82 mm, I.D. 0.65 mm, Fishman Corporation, Hopkinton, MA, USA). These needles were connected to external electromagnetic valves via resistive steel capillaries (O.D. 400 μm, I.D. 205 μm, length 100 cm, Mifam, Milanowek, Poland), assisted by sections of Tygon tubing (~2 cm, O.D. 0.91 mm, I.D. 0.25 mm, Ismatec, Wertheim, Germany). The use of steel capillaries with high hydrodynamic resistance enabled precise control of substance flow, allowing for iterative operations, such as on-demand droplet generation. Other inlets and outlets in the system were established using 50 cm Teflon fluorinated ethylene propylene (FEP) tubing (O.D. 1.6 mm, I.D. 0.8 mm, Bola).

### 3.3. Oil-Droplet Generation and System Operation

To meet the system requirements, oil droplets (dispersed phase) were generated within two T-junctions (see Figure 1A,C) on the microfluidic chip as needed. This was achieved by flushing both sides of the Snapwell insert with preset fluids. The initiation of oil-droplet generation coincided with the closure of the electromagnetic valves responsible for dispensing KHS into the cell chamber. Subsequently, the valves supplying oil to the microfluidic system were opened for 20 μs. In our system, this duration allowed us to produce oil droplets with a length 20 times the width of the channel, i.e., $L_{d}=8\;mm$ (volume: $V_{d}=1.28\;\mu L$), ensuring a precision of 0.8% in administering droplet volumes. According to the literature, droplets of this length exhibit a constant velocity relative to the continuous phase despite minor fluctuations in their dimensions [24]. To verify this, we scattered droplets with lengths ranging from 7 to 9 mm and observed no changes in the mobility (see Figure 1D). Mobility (β) is conventionally defined as the velocity of the droplet phase relative to the average velocity of the continuous phase. Measurements were conducted for all solutions used and revealed no differences in droplet mobility due to the change in the continuous phase (see Figure 1E). Furthermore, no fluctuations in oil-droplet velocity relative to the continuous phase were noted within the range of small capillary numbers, similar to the measured actual water flows through the cells. The obtained mobility value was β = 1.03 ± 0.013 within the capillary number range of $Ca=\left\{{2*10}^{-4};10^{-8}\right\}$ for all employed fluids: KHS1, KHS2, and KHS3. 

### 3.4. Droplet Tracking and Analysis

The generated oil droplets traversed two parallel arms of the microfluidic system, their movement tracked by a 2D CMOS chip camera (uEye, USB UI-3140CP Rev.2). The camera, focused on the centerline of the microchannels, captured images at 169 Hz in real-time, facilitating subsequent analysis. Droplet lengths were measured by analyzing the fore- and rear-caps, which produced distinguishable negative peaks in pixel intensity recorded by the camera. The Poisson distribution best fit these troughs, and this distribution was employed in all experiments. The estimated error in droplet length measurement was below 1%.

Knowing the change in the position of the droplets at known time intervals (calculated from the camera sampling rate ~0.006 s) and the dependence of the droplet mobility on the flow rate of the continuous phase, the volume of liquid flowing through the epithelial cell layer was determined.

### 3.5. Integration with Control and Analysis Software

The camera, electromagnetic valves, and digital manometers were seamlessly integrated into a computerized system using National Instruments cards (NI PCIE-6320) for optimal control and data acquisition. Leveraging a custom-written LabWindows software interface (LabWindows/CVI 2019), our experimental setup afforded a multifaceted range of functionalities. Specifically, this system enabled us to do the following: (i) meticulously monitor the real-time pressure of the continuous liquid phases (Krebs–Henseleit solutions) and the oil-droplet phase, providing invaluable insights into the dynamic fluidic conditions within the microfluidic device; (ii) precisely create droplets on demand through the seamless control of electromagnetic valves, allowing for iterative and controlled experimentation; (iii) analyze high-frequency images acquired from the camera at a rapid sampling rate of 169 Hz, providing detailed insights into droplet dynamics and behavior; and (iv) extract the velocity of the generated droplets, facilitating a quantitative understanding of fluid flow through the epithelial cell monolayers. This integration of advanced hardware components with sophisticated software control not only ensured precise experimental execution but also facilitated real-time data analysis, contributing to the accuracy and efficiency of our measurements.

### 3.6. Key Features of Designed System

Our innovative microfluidic system incorporates key features tailored to address the rigorous demands of epithelial cell studies, encompassing the following attributes:

- Contamination prevention: The architecture is specifically engineered to prevent cross-contamination between the apical and basolateral sides of cell layers. This crucial design aspect ensures the integrity of experiments, maintaining the purity of the cellular microenvironment.
- Real-time velocity measurement: The system enables the rapid and real-time measurement of droplet velocity immediately following fluid exchange on both sides of the cell chamber. This capability provides researchers with dynamic insights into the fluid transport dynamics across epithelial cell monolayers.
- Reproducible droplet generation: The microfluidic device excels in the generation of droplets with precisely reproducible volumes. This high level of precision, down to minuscule fluid volumes, enhances the reliability and accuracy of experimental outcomes, contributing to the robustness of the findings.
- Droplet routing and positioning: The system is equipped to effectively route and position droplets on the chip for continuous monitoring. This feature ensures that droplets can be tracked and analyzed with precision, facilitating detailed investigations into fluid flow patterns and transport phenomena across the epithelial cell layers.

These integral features collectively position the microfluidic system as an advanced tool for studying epithelial transport, providing researchers with unparalleled control, precision, and real-time monitoring capabilities. The system’s capabilities open avenues for a profound comprehension of intricate physiological processes, holding promise for uncovering novel insights into epithelial cell behavior.

## 4. Results

### 4.1. Water Transport Measurements in Chloride Gradients

**Figure 2:**
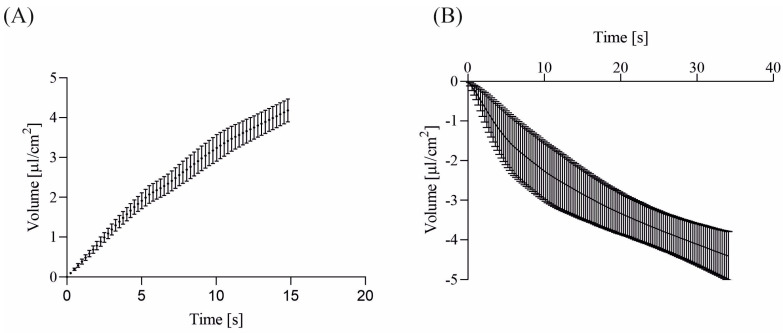
The figure delineates water transport across the 16HBE14σ cell monolayer under the influence of chloride gradients. Panel (**A**) demonstrates water transport across cell monolayers with the apical chamber filled with a low-chloride solution (KHS3) and the basolateral chamber filled with a high-chloride solution (KHS1). In Panel (**B**), water transport occurs in the reversed chloride gradient, with the apical chamber filled with a high-chloride solution (KHS1) and the basolateral chamber filled with a low-chloride solution (KHS3). The data are presented as mean ± SD (*n* = 5). Positive values indicate basolateral to apical water flux, while negative values indicate apical to basolateral water flux.

The water transport measurement system was employed to gauge transepithelial water flow within ionic gradients. In reaction to the transepithelial chloride gradient, water traversed polarized epithelial cell monolayers from higher to lower chloride concentrations. The pace of water transport across the cell monolayers was notably swifter when the induced gradient prompted chloride to flow from the basolateral to apical side as opposed to the reverse direction (refer to Figure 2).

### 4.2. Water Transport Measurements in Sodium Gradients

**Figure 3:**
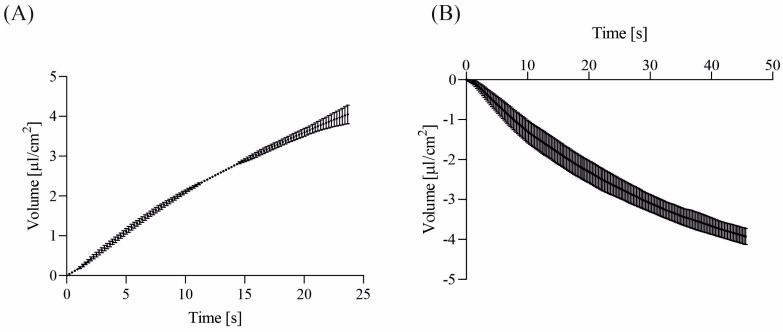
Water transport across 16HBE14σ cell monolayers was assessed under sodium gradients. (**A**) Water transport was examined in cell monolayers with the apical chamber filled with a low-sodium solution (KHS2), while the basolateral chamber contained a high-sodium solution (KHS1). (**B**) Conversely, water transport was studied in cell monolayers with a reversed sodium gradient. In this case, the apical chamber was filled with a high-sodium solution (KHS1), and the basolateral chamber contained a low-sodium solution (KHS2). The data are presented as mean ± SD (*n* = 4). Positive values indicate basolateral to apical water flux, while negative values denote apical to basolateral water flux.

In our exploration, we conducted a series of experiments involving sodium, wherein we observed the movement of water down the sodium concentration gradient. The kinetics of water transport exhibited notable differences under two distinct experimental conditions. Intriguingly, akin to observations made with chloride gradients, the rate of water transport proved to be swifter when a high-sodium solution (KHS1) was introduced on the basolateral side with a concurrent introduction of a low-sodium solution (KHS2) on the apical side of cell monolayers (refer to Figure 3A) as opposed to the reverse scenario (see Figure 3B).

Of particular interest is the time interval between the 11th and 14th seconds in Figure 3A, where the measured values show minimal error. Regrettably, despite our scrutiny, we have been unable to propose a reasonable hypothesis to account for this intriguing observation.

## 5. Discussion

**Figure 4:**
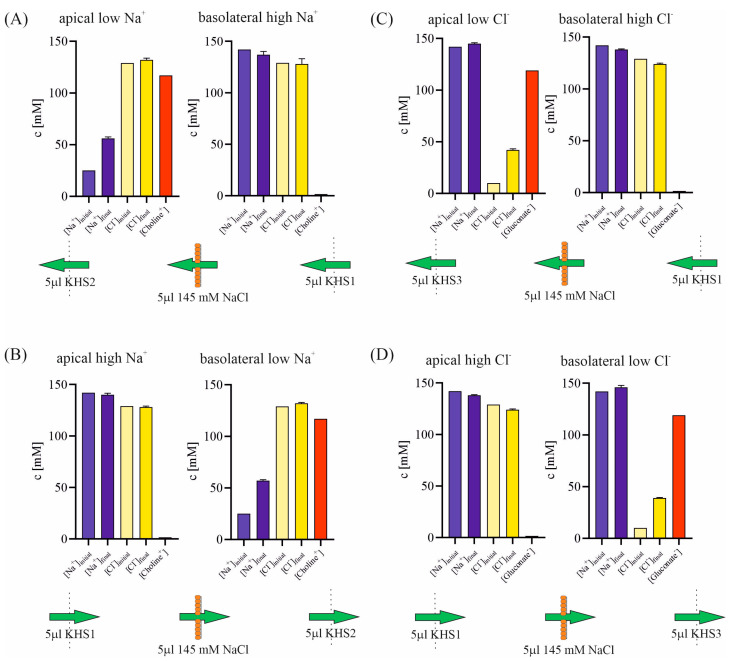
The study investigated the initial and final concentrations of sodium, chloride, choline, and gluconate ions on either side of the cell layer, as well as the direction of solution transport across epithelial cell monolayers. The experiments were conducted under the influence of different gradients: (**A**) basolateral-to-apical sodium gradient; (**B**) apical-to-basolateral sodium gradient; (**C**) basolateral-to-apical chloride gradient; (**D**) apical-to-basolateral chloride gradient. Final concentrations were determined in the 5th minute after the cessation of solution flow.

In previous investigations, we assessed the multiple ion transport across the 16HBE14σ cell line utilizing ion-selective electrodes [1,2,3] within isoosmotic media characterized by variations in sodium or chloride concentration. The observed transport was found to be electroneutral, with no discernible change in transepithelial potential. Notably, the pH remained constant throughout the experiment. Despite these seemingly straightforward conditions, the interpretation of our experimental findings presented unexpected challenges, as depicted in Figure 4.

During the ionic gradients experiment, a notable increase in the concentration of either sodium or chloride is observed on the lower-concentration side, as illustrated in Figure 4. However, this elevation on the low-concentration side does not correspond to a decrease in sodium or chloride concentration on the high-concentration side. Furthermore, there is no significant alteration in the concentration of the counterion (chloride in experiments involving sodium gradients and sodium in experiments with chloride gradients) on either side of the cell layer. In light of the electroneutral nature of the transport, our interpretation of the results suggests a transport of 5 µL of 145 mM NaCl solution across the cell layer. Are there alternative explanations for our experimental findings? Since there is no pH change, the influence of H^+^ or OH^−^ ion transport appears negligible. Could the transport of choline or gluconate ions offer an explanation for our experimental results?

Both choline and gluconate ions have the capability to traverse the epithelium through organic cation or organic anion transporters [25,26,27]. However, it is crucial to note that transporter molecules are three to five orders of magnitude less effective than ion channels in ion transportation. For instance, choline transport across Caco-2 cells is reported as 2800 pmol/mg protein/10 min [28], while in our experiment, sodium is transported across the cell monolayer at a speed of 290 µmol/mg protein/5 min—underscoring a five-orders-of-magnitude difference. Consequently, in electrophysiological measurements, it is conventionally assumed that the transport of large organic ions is negligibly small compared to inorganic ion transport. Even if the exchange of choline for sodium (or gluconate for chloride) were feasible, we would not observe a decrease in sodium concentration on the high concentration side, and such an exchange would not induce fluid flow across the cell monolayer.

Our direct water transport measurements, as depicted in Figure 2 and Figure 3, reveal that approximately 25% of the chamber volume (20 µL) flows across the cell monolayer within a minute—aligning with our semiquantitative calculations based on ion-selective measurements [1,3]. This supports the hypothesis of a flux of 145 mM NaCl solution across the epithelial layer in ionic gradient solutions. The fluid fluxes for various experimental conditions are summarized in Table 1.

After half a century of studying epithelial transport, the typical experimental procedure involved applying an ionic gradient across the epithelium and introducing modifiers of the transporting molecules. Our results indicate that the introduction of an ionic gradient forcibly alters the transport properties of the epithelia. In the case of large-volume Ussing chambers, this change appears to be permanent. However, in our case, utilizing a very-small-volume chamber, the transport reaches a new equilibrium after just a few minutes.

### Self-Regulating Mechanism of Water Transport in Bronchial Epithelia—The Hypothesis

The bronchial epithelium plays a crucial role in transporting water and CO_2_ out of the organism, adapting to various environmental conditions such as temperature and humidity [29,30]. Maintaining an optimal volume of airway surface liquid (ASL) is essential, as excessively high fluid levels can hinder CO_2_ transport, while insufficient levels can lead to viscosity issues. The intricate regulation of water transport in the bronchial epithelium suggests the presence of a finely tuned mechanism, possibly located at the apical face of the epithelium. Despite this, the exact nature of this mechanism remains elusive.

While mechanical stress has been proposed as a potential trigger [31], our measurements indicate fluid flows across the epithelium in both directions even in the absence of mechanical stress, with isoosmotic solutions present on both sides. Another hypothesis suggests the involvement of a chloride sensor [32]. However, our experiments demonstrate rapid fluid flow despite identical chloride concentrations on either side of the epithelium.

Furthermore, the concentration of signaling molecules, such as ATP, dissolved in the ASL appears to influence water transport. Our findings reveal that water secretion decreases ATP concentration in the ASL, altering the direction of fluid flow, while absorption increases ATP concentration [33,34]. Surprisingly, when we conducted experiments without ATP (or other specific molecules) during medium exchange, robust fluid flow occurred in both directions. Consequently, we have reconsidered our model of water flow through the bronchial epithelium, proposing that no specific trigger switches the direction of water flow. Instead, we posit that simple electrochemical considerations are adequate to elucidate the self-regulating mechanism governing water transport in the ASL.

In our preceding article, we put forth a conceptual framework elucidating the intricate mechanism of water transport across bronchial epithelium [1]. Our proposition posits the involvement of the CFTR channel, apical Na/H and Cl/HCO_3_ exchangers, and paracellular sodium ion transport in the regulation of airway surface liquid (ASL) volume. Specifically, we hypothesized that the interplay of chloride ions, paracellularly transported sodium ions, and bicarbonate anions orchestrates the delicate balance of water movement.

When chloride ions, along with paracellularly transported sodium ions, traverse the epithelial cell, they induce a flow of water from the cell into the ASL. Conversely, the secretion of bicarbonate anions triggers a reaction with protons, resulting in the formation of weakly dissociated carbonic acid with low osmotic pressure. This phenomenon prompts water to move from the ASL into the cell. It is noteworthy that the CFTR channel plays a pivotal role in this process, facilitating the transport of both chloride and bicarbonate anions with a selectivity ratio of k_Cl/HCO3_ = 4 [35,36].

The direction of ion transport, whether outward from or inward to the cell, is contingent upon the energy of the process [37]. For each ion species, the electrochemical potential difference between both sides of the cell membrane can be described by the following equation:

$$∆\mu_{i}=RT*ln\frac{c_{i,ext}}{c_{i,cell}}+zF*\left(\varphi_{ext}-\varphi_{cell}\right)$$

where the concentration of the ion species ‘*i*’ is denoted by ‘*c*’, with subscripts ‘*cell*’ and ‘*ext*’ representing the cytoplasm and external medium, respectively. The symbols *R*, *T*, *F*, and z correspond to the gas constant, temperature, Faraday constant, and ion charge, respectively. Typically, *φ_ext_* ≡ 0 is adopted as the reference potential, and results are presented in (kJ/mol).

When Δ*µ_i_* > 0, ions migrate into the cell, and when Δ*µ_i_* < 0, they exit the cell. Equilibrium is reached when Δ*µ_i_* = 0. Both channels and exchangers exhibit bidirectional functionality, influenced by the disparity in electrochemical potentials of the involved ions (in this case, Δ*µ_Cl_* and Δ*µ_HCO3_*).

**Figure 5:**
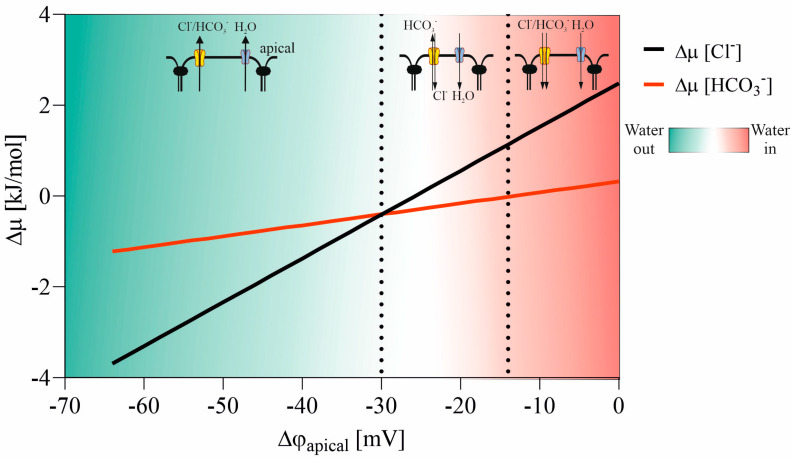
Ionic transport direction in the CFTR channel relative to apical potential difference.

Ion movement through the CFTR channel is contingent upon both the electric potential difference across the apical membrane (Δ*φ_ap_*) and the ion composition of the airway surface liquid (ASL), or external medium, and the cytoplasm. Assuming [Cl]_cell_ = 40 mM [35], [HCO_3_]_cell_ = 15 mM [36], [Cl]_ext_ = 108 mM [38], and [HCO_3_]_ext_ = 25 mM, one can compute chloride and bicarbonate ion transport through the CFTR channel as a function of the potential difference across the apical membrane of the cell, as illustrated in Figure 5.

The anions flow out from the cell through the CFTR channel, reducing Δφ_ap_. The equilibrium for water movement across the apical membrane is achieved at Δφ_ap_ = −30 mV (for a chloride concentration of [Cl]_cell_ = 40 mM). Below –30 mV, the CFTR channel begins to act as an exchanger, allowing chloride ions to enter the cytoplasm and bicarbonate anions to flow out (see Figure 5). Reaching Δφ_ap_ = –30 mV alters the direction of water flow across the apical membrane. In bronchial epithelium, it is expected that Δφ_ap_ will oscillate around the equilibrium potential value. Several experiments measuring apical potential difference and cytoplasmic chloride concentration have been conducted. Clarke et al. [39] determined Δφ_ap_ = −17 ± 1.5 mV and cytoplasmic chloride activity [Cl]_cell_ = 56 mM in a medium with [Cl]_ext_ = 125 mM. Our model suggests that under such conditions, the equilibrium potential would be Δφ_ap_ = –22 mV, a value close to the measured result. This indicates that simple electrochemical considerations are adequate to explain self-regulating water transport through epithelium in healthy subjects.

**Figure 6:**
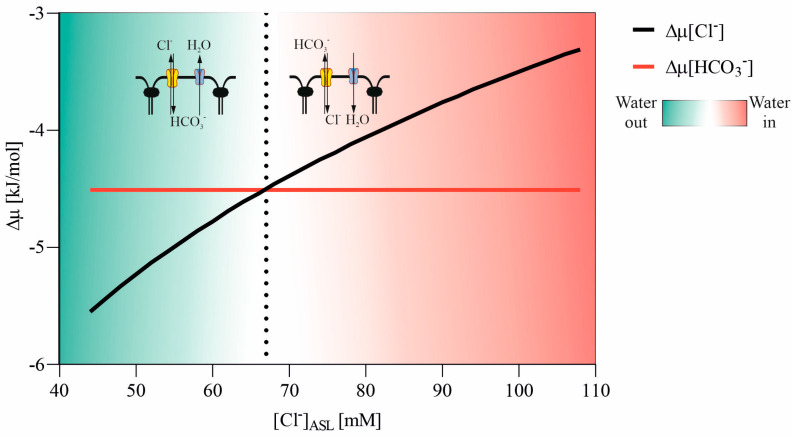
Directional preference of the Cl/HCO_3_ exchanger in response to varied chloride concentrations in the airway surface liquid (ASL) or medium.

In cystic fibrosis (CF), the dysfunctional CFTR channel cannot influence water transport balance. Δφ_ap_ is not reduced by anion outflow via the CFTR channel, but the apical fluid’s chloride concentration decreases due to the absence of CFTR-mediated transport. Figure 6 illustrates how the anion exchanger in the apical membrane responds to changes in ASL chloride concentration. For [Cl] = 67 mM in the external medium (ASL), the direction of anion transport shifts from “chloride in and bicarbonate out” (with water flowing into the cell) to “bicarbonate in and chloride out,” causing water to flow out of the cell. A new water balance transport is established, and the chloride concentration in ASL oscillates at much lower values in CF compared to healthy subjects. Hull et al. [38] measured ASL chloride concentration in CF subjects and found [Cl]_CF_ = 77 ± 7 mM, very close to the value predicted by our model. Thus, the anion exchanger at the apical membrane plays a significant role in water transport across the epithelium in CF subjects.

In the sweat gland and nasal epithelia, it is imperative to highlight that the mechanism of salt reabsorption relies on the activity of ENaC channels. These channels facilitate the transportation of sodium into the cytoplasm, consequently reducing Δφ_ap_ to exceptionally low values. At Δφ_ap_ = −14 mV, the CFTR channel plays a pivotal role by transporting anions from the sweat to the cell, as illustrated in Figure 5. In individuals with cystic fibrosis (CF), the activation of ENaC leads to a decline in φ_ap_, which remains uncompensated by anion transport. Consequently, the transepithelial potential, also known as sweat gland potential or nasal potential, attains more negative values than observed in healthy subjects, aligning with the expectations outlined in the model, as demonstrated in previous studies [40,41].

## 6. Conclusions

In the bronchial epithelium of 16HBE14σ cells, sodium ions traverse through both paracellular and transcellular pathways, whereas chloride ions exclusively utilize the transcellular route. Notably, the swift transport of Na^+^ and Cl^−^ manifests bidirectionally across the cell monolayer, even under isoosmotic conditions on both sides, coinciding with a flow of 145 mM NaCl.

The fluid flux across the 16HBE14σ cell monolayer can peak at 0.9 µL/cm^2^∙s, resulting in a rapid one-second ascent of the airway surface liquid (ASL) by 0.9 µm in the sodium ion gradient. Conversely, the flux can descend to as low as 8 × 10^−4^ µL/cm^2^∙s, equivalent to a gradual ASL rise of 3 µm over one hour.

In individuals with normal respiratory function, the regulation of water flux into and out of the ASL is orchestrated by the CFTR channel, achieving equilibrium when the apical potential approximates Δφ_ap_ = –30 mV. Conversely, in the context of cystic fibrosis, the modulation of water transport is overseen by an anion exchanger residing on the apical membrane, establishing equilibrium at [Cl] = 67 mM in the ASL.

## Figures and Tables

**Table 1:** The initial flow and flux values were measured in the 16HBE14σ cell line. All solutions used were isoosmotic. Mean flux values (µL/cm^2^∙s) were determined within the first 5 s of the experiment. The surface area of the Snapwell cup used was 1.1 cm^2^. For symmetrical KHS1 solutions, the reported values represent the mean fluid movement over the course of 1 h. Positive values indicate a flow from the basolateral to the apical side of the cell layer, while negative values denote a flow in the opposite direction.

Apical Medium	Basolateral Medium	$\mathrm{Stream}\;(\mu L/{\mathrm{cm}}^{2}\cdot$s)
KHS1	KHS2 (low sodium)	−0.909 ± 0.035
KHS2 (low sodium)	KHS1	+0.201 ± 0.013
KHS1	KHS3 (low chloride)	−0.346 ± 0.036
KHS3 (low chloride)	KHS1	+0.261 ± 0.125
KHS1	KHS1	+0.000813 ± 0.000066

## Data Availability

The data presented in this study are available upon request from the corresponding author.
